# Addressing the Second Delay in Saving Mothers, Giving Life Districts in Uganda and Zambia: Reaching Appropriate Maternal Care in a Timely Manner

**DOI:** 10.9745/GHSP-D-18-00367

**Published:** 2019-03-11

**Authors:** Thandiwe Ngoma, Alice R. Asiimwe, Joseph Mukasa, Susanna Binzen, Florina Serbanescu, Elizabeth G. Henry, Davidson H. Hamer, Jody R. Lori, Michelle M. Schmitz, Lawrence Marum, Brenda Picho, Anne Naggayi, Gertrude Musonda, Claudia Morrissey Conlon, Patrick Komakech, Vincent Kamara, Nancy A. Scott

**Affiliations:** a Right to Care-Zambia, Lusaka, Zambia.; b Baylor College of Medicine Children's Foundation-Uganda, Kampala, Uganda.; c Division of Reproductive Health, U.S. Centers for Disease Control and Prevention, Atlanta, GA, USA.; d Harvard T.H. Chan School of Public Health, Boston, MA, USA.; e Department of Global Health, Boston University School of Public Health, and Section of Infectious Diseases, Department of Medicine, Boston Medical Center, Boston, MA, USA.; f School of Nursing, University of Michigan, Ann Arbor, MI, USA.; g U.S. Centers for Disease Control and Prevention, Lusaka, Zambia. Now retired.; h Infectious Diseases Institute, College of Health Sciences, Makerere University, Kampala, Uganda.; i Africare, Washington, DC, USA.; j Africare, Lusaka, Zambia.; k Bureau for Global Health, U.S. Agency for International Development, Washington, DC, USA.; l Division of Global HIV and TB, U.S. Centers for Disease Control and Prevention, Kampala, Uganda.

## Abstract

**Background:**

Between June 2011 and December 2016, the Saving Mothers, Giving Life (SMGL) initiative in Uganda and Zambia implemented a comprehensive approach targeting the persistent barriers that impact a woman's decision to seek care (first delay), ability to reach care (second delay), and ability to receive adequate care (third delay). This article addresses how SMGL partners implemented strategies specifically targeting the second delay, including decreasing the distance to facilities capable of managing emergency obstetric and newborn complications, ensuring sufficient numbers of skilled birth attendants, and addressing transportation challenges.

**Methods:**

Both quantitative and qualitative data collected by SMGL implementing partners for the purpose of monitoring and evaluation were used to document the intervention strategies and to describe the change in outputs and outcomes related to the second delay. Quantitative data sources included pregnancy outcome monitoring data in facilities, health facility assessments, and population-based surveys. Qualitative data were derived from population-level verbal autopsy narratives, programmatic reports and SMGL-related publications, and partner-specific evaluations that include focus group discussions and in-depth interviews.

**Results:**

The proportion of deliveries in any health facility or hospital increased from 46% to 67% in Uganda and from 63% to 90% in Zambia between baseline and endline. Distance to health facilities was reduced by increasing the number of health facilities capable of providing basic emergency obstetric and newborn care services in both Uganda and Zambia—a 200% and 167% increase, respectively. Access to facilities improved through integrated transportation and communication services efforts. In Uganda there was a 6% increase in the number of health facilities with communication equipment and a 258% increase in facility deliveries supported by transportation vouchers. In Zambia, there was a 31% increase in health facilities with available transportation, and the renovation and construction of maternity waiting homes resulted in a 69% increase in the number of health facilities with associated maternity waiting homes.

**Conclusion:**

The collective SMGL strategies addressing the second delay resulted in increased access to delivery services as seen by the increase in the proportion of facility deliveries in SMGL districts, improved communication and transportation services, and an increase in the number of facilities with associated maternity waiting homes. Sustaining and improving on these efforts will need to be ongoing to continue to address the second delay in Uganda and Zambia.

## Abstract (precis)

The Saving Mothers, Giving Life initiative employed 2 key strategies to improve the ability of pregnant women to reach maternal care: (1) increase the number of emergency obstetric and newborn care facilities, including upgrading existing health facilities, and (2) improve accessibility to such facilities by renovating and constructing maternity waiting homes, improving communication and transportation systems, and supporting community-based savings groups. These interventions can be adapted in low-resource settings to improve access to maternity care services.

## INTRODUCTION

Global guidelines developed by the World Health Organization (WHO) recommend that women deliver at facilities with the capacity to manage emergency obstetric and newborn care (EmONC) as a strategy to improve maternal and newborn mortality.^1^ WHO identified a set of medical interventions or signal functions that address the direct causes of maternal death, with 7 of these interventions defining basic emergency obstetric and newborn care (BEmONC).^2^ However, despite the recommendation, persistent barriers affect a woman's decision to seek care (first delay), ability to reach care (second delay), and ability to receive adequate maternal health services (third delay), as outlined in the Three Delays Model developed in 1994 by Thaddeus and Maine.^3^ While this conceptual framework was first developed to understand health care decision making and access to care for complications during delivery, it has been adapted to understanding decision making and access around location of normal delivery as well.^4^

**FIGURE 1:**
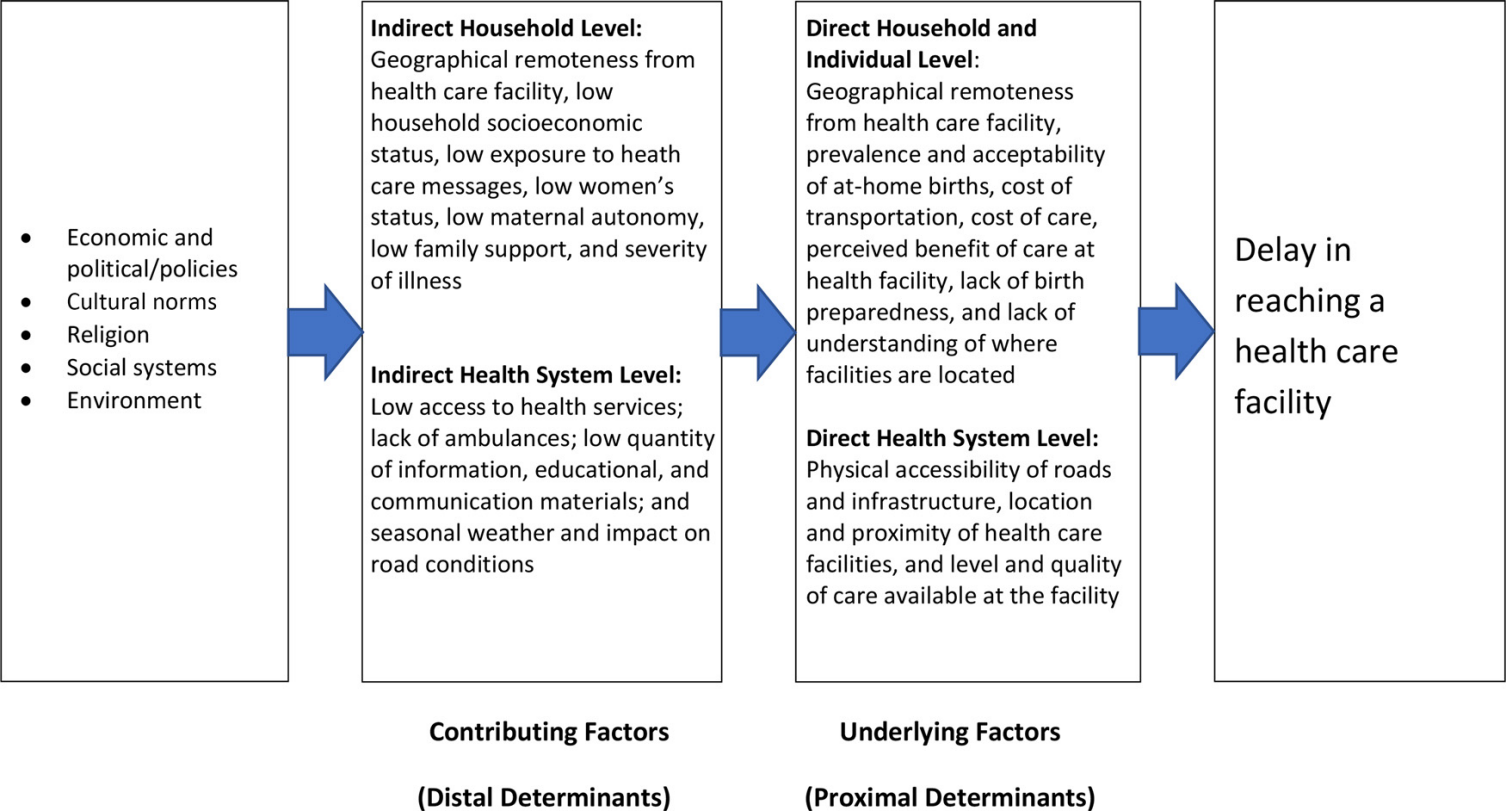
Second Delay: Timely Access to Health Care

The second delay—the delay in the ability to reach care—is fueled by factors that both directly and indirectly influence a woman's ability to reach care, including long distance to facilities, geographical barriers, poor road infrastructure, lack of transportation options, poor communication, and costs associated with delivery such as transportation and supplies (Figure 1).^5^^,^^6^ In rural Ghana, a recent study indicated that travel time was inversely associated with facility delivery even when facilities had improved their capacity to handle obstetric emergencies.^7^^,^^8^ Other barriers include high costs of available transportation or supplies,^4^ lack of a clear birth plan^9^ or not departing for the facility with sufficient time before labor,^10^^–^^12^ and limited access to financial resources.^13^

### Interventions to Reduce the Second Delay

Intervention approaches to address these barriers include those that target the health system (supply side) and those targeting pregnant women (demand side).^14^ Known interventions designed to address health system barriers include strengthening referral systems and transportation, improving communication tools, establishing maternity waiting homes, and developing or strengthening community-based systems to escort women to facilities.^15^^,^^16^ Activities targeting pregnant women include community-based linkages to health facilities, financing mechanisms to prepare for facility delivery, and birth preparedness. Demand-side financing mechanisms, such as cash transfers and vouchers, have been introduced in several countries and have been effective in increasing the utilization of maternity care.^17^^,^^18^ A recent review of cash transfers and vouchers found the strongest effect for birth with a skilled birth attendant was observed for those who used vouchers for maternity services.^19^ However, reflecting the significance of supply-side barriers, women who lived long distances from facilities and had poor access to transportation still faced challenges to accessing services.

### SMGL Context

According to the 2011 Uganda Demographic Health Survey, 58% of births in the previous 5 years took place in a health facility.^20^ The 2013–2014 Zambia Demographic Health Survey found that 67% of deliveries in the previous 5 years were in a health facility. Health facilities being too far and a lack of transportation were cited as reasons for home delivery by 1 in 3 women who delivered at home.^21^ In both Uganda and Zambia, women from urban areas were more likely to deliver at a health facility compared with those in rural areas. In Uganda, 89% of women in urban areas and 53% of women in rural areas delivered at a facility. In Zambia, 89% of women in urban areas delivered at a facility compared with 56% in rural areas.

As part of its comprehensive strategy to address maternal mortality, the Saving Mothers, Giving Life (SMGL) initiative,^22^ in partnership with the governments of Uganda and Zambia, implemented a package of interventions specifically targeting the second delay. A mix of interventions was implemented under 2 broad strategies: (1) increasing the number of EmONC facilities and (2) improving the accessibility of EmONC facilities (Table 1). For each intervention, implementing partners monitored and evaluated their efforts to contribute to the collective understanding of the overall impact of the SMGL initiative. This article provides an overview of the SMGL interventions that focused on the second delay in Uganda and Zambia, and uses programmatic data to describe the outputs and outcomes of these interventions.

**TABLE 1.** Saving Mothers, Giving Life Strategies and Interventions to Reduce the Second Delay, 2011–2016

SMGL Strategies and Approaches	Country-Specific Interventions	
	Uganda	Zambia
**Strategy 1. Decrease distance to skilled birth attendance by increasing the number of EmONC facilities**		
Establish additional EmONC facilities and strengthen existing facilities to provide the following services 24 hours per day, 7 days a week, for all pregnant women in the district: Clean and safe basic delivery services Quality HIV testing Counseling and treatment (for woman, partner, and baby as appropriate) Essential newborn care 24-hour availability of staff capable of managing delivery complications When needed, timely facilitated referral to higher-level facility	Upgraded infrastructure to a sufficient number of public and private facilities in appropriate geographic locations and provided necessary equipment and commodities for EmONC service delivery Hired midwives, medical officers, and anesthetists Trained medical officers, anesthetists, midwives, and nurses in EmONC Provided on-site mentorship of health facility teams using protocols	Upgraded infrastructure and provided necessary equipment to provide services for pregnant women in public and private facilities in appropriate geographic locations Hired a sufficient number of skilled birth attendants and midwives Trained doctors, midwives, and anesthetists in EmONC and the Electronic Logistic Management Information System Provided on-site mentorship of health facility staff using protocols, forms, and drills
**Strategy 2. Improve accessibility of EmONC facilities**		
Create a communication and transportation referral system that operates 24 hours per day, 7 day per week, and: Is consultative, protocol-driven, quality-assured, and integrated (public and private) Ensures that women with complications reach emergency services within 2 hours Includes buying ambulances, motorcycles, motorbikes, and communication equipment like 2-way radios Provides or renovates, where appropriate, temporary lodging in maternity waiting homes for women with high-risk pregnancies or who live more than 2 hours travel time to an EmONC facility Provides service delivery vouchers and vouchers for transport to basic delivery care facilities and referral to higher-level facilities Forms district-level transport committees to improve referral	Created district transportation committees to improve coordination of ambulances for referrals Provided service and transportation vouchers to women for transportation to facilities nearest to them and access to antenatal care, delivery, and postnatal care services at the facilities Trained village health teams to encourage birth preparedness and escort women to the facility Procured ambulances to facilitate transportation for referral Renovated maternity waiting homes	Repaired and procured 2-way radios where needed Procured ambulances and motorcycle ambulances; strengthened district transportation committees; and ensured strategic placement of ambulances Renovated and constructed maternity waiting homes Strengthened district transportation committees to improve coordination of ambulance services Trained Safe Motherhood Action Groups to encourage birth preparedness and escort women to the facility Established village-level savings programs for pregnant women to encourage better planning for delivery

Abbreviations: EmONC, emergency obstetric and newborn care; SMGL, Saving Mothers, Giving Life.

**FIGURE 2:**
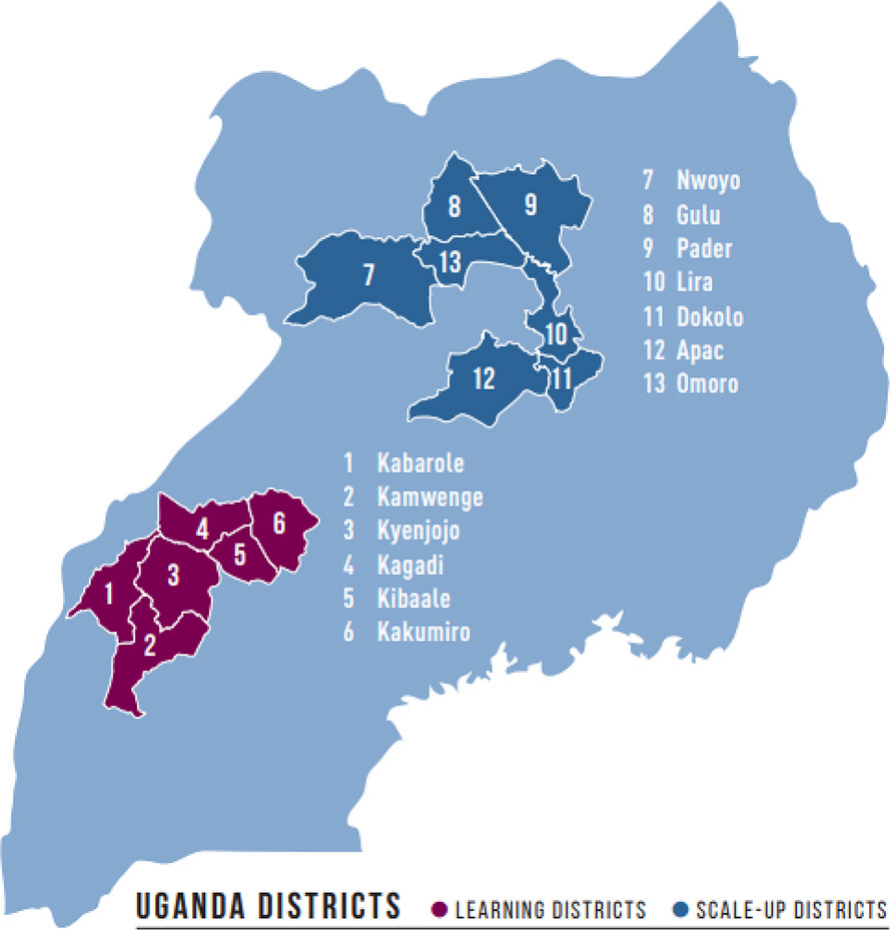
SMGL Learning and Scale-Up Districts in Uganda Source: Adapted from Saving Mothers, Giving Life. Results of a Five-Year Partnership to Reduce Maternal and Newborn Mortality: Final Report 2018. http://www.savingmothersgivinglife.org/docs/smgl-final-report.pdf. Accessed December 18, 2018.

**FIGURE 3:**
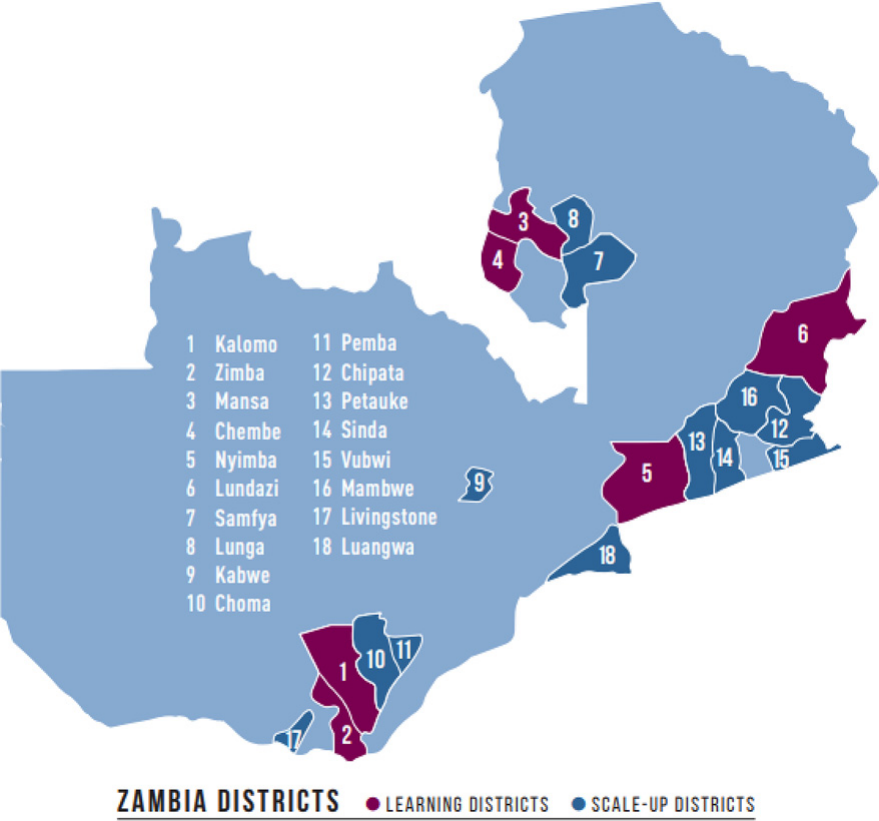
SMGL Learning and Scale-Up Districts in Zambia Source: Adapted from Saving Mothers, Giving Life. Results of a Five-Year Partnership to Reduce Maternal and Newborn Mortality: Final Report 2018. http://www.savingmothersgivinglife.org/docs/smgl-final-report.pdf. Accessed December 18, 2018.

The SMGL initiative in both Uganda and Zambia operated within 3 phases: Phase 0—design and start-up (June 2011 to May 2012); Phase 1—proof of concept (June 2012 to December 2013); and Phase 2—scale-up and scale-out (January 2014 to October 2017). During Phase 2, SMGL expanded its presence in Uganda from 4 districts to 13 districts (Figure 2), and in Zambia from 6 to 18 districts (Figure 3). For this article, we focused on the initial districts selected in Phase 0 (before the Phase 2 expansion) from June 2011 to December 2017. Implementing districts were contiguous in Uganda, whereas the Zambian districts were spread out across 3 provinces, with 2 in Eastern Province, 1 in Southern Province, and 1 in Luapula Province in the northern region.

In both Uganda and Zambia, all SMGL implementing districts were predominantly rural, with geographic distance and mountainous terrain playing a major role in the accessibility of EmONC services. Most roads were packed dirt, with only a handful of paved roads primarily between main towns and the district capitals. Dense forest coverage and low population density characterized large portions of the implementation districts in both countries. Further details on the scope of the program and district and facility characteristics have been described elsewhere.^23^

## METHODS

We reviewed both quantitative and qualitative data to describe the implementation of SMGL interventions targeting the second delay and to evaluate collective outcomes. Partners collected program-specific data as well as indicators that were harmonized across a broader SMGL monitoring and evaluation strategy. In both countries, the U.S. Centers for Disease Control and Prevention country office oversaw partner-specific data collection and evaluation activities and each implementing partner had data quality control measures in place for data collection and data entry. We also reviewed partner-specific programmatic data and data from systematic evaluations. In both countries, SMGL partners carried out formative research to understand the country context and determine which specific factors influenced the practice of key behaviors before, during, and after delivery.

### Quantitative Data and Analytic Methods

Pregnancy outcome monitoring data in facilities, health facility assessments, and population-based data were the primary quantitative data sources used to assess programmatic outcomes related to strategies addressing the second delay across all SMGL-supported districts. Details on data collection methods have been published elsewhere.^24^

#### Pregnancy Outcomes Monitoring Data

Implementing partners collected routine data on 31 SMGL indicators from the labor and delivery and other in-patient registers in facilities in SMGL-supported districts. Baseline indicators were calculated from data collected during Phase 0 between June 2011 and May 2012, and endline indicators were calculated from data collected between January and December 2016. We then calculated the relative change between the baseline and endline indicators. To compare the baseline and endline results for significant differences, we used the McNemar's test, which is appropriate for dichotomous responses for matched pairs of data at different time points.

#### Health Facility Assessments

Health facility assessments were conducted at baseline (late 2011 in Zambia and early 2012 in Uganda) and at endline (2017) in all health facilities in SMGL-supported districts. We used these data to document the status of health facilities and their availability of lifesaving emergency obstetric interventions at the time of the assessment. In this article, we present results compiled from facilities that maintained delivery capacity from baseline to endline—105 in Uganda and 110 in Zambia. The assessments were aligned with the WHO criteria for basic (BEmONC) and comprehensive emergency obstetric and newborn care (CEmONC)^2^ and included questions about facility infrastructure, staffing, ability to perform signal functions,^2^ stock-outs of key medications required for the management of complications, and referral system components including transportation and communication.

We classified facilities as EmONC if, in the previous 3 months, they performed all 7 of the signal functions for BEmONC and all 9 for CEmONC at the time of the assessment, and non-EmONC if they were not capable of performing all of the BEmONC signal functions.

#### Population-Based Data

Population-level household surveys (Reproductive Age Mortality Study in Uganda and SMGL Census in Zambia) were conducted in 2012 and 2017.^24^ We combined household data with the health facility routine monitoring data and health facility assessment data, to calculate the proportion of facility deliveries, stratified by EmONC capacity, at baseline and endline. The facility delivery rate was calculated using the number of deliveries verified to have occurred in an SMGL-affiliated facility divided by the estimated number of live births in the SMGL districts at each time point. The number of births was estimated by applying crude birth rates (derived from the age-specific fertility rates among women of reproductive age enumerated in 2013 in Uganda districts, and derived from the 2010 national census in Zambia) to the baseline and endline district population. We calculated the relative change in facility deliveries between baseline and endline, assuming some variation in error or measurement. To test for significance, *z* scores based on the normal approximation to the binomial distribution were used to calculate *P* values.

### Qualitative Data and Analytic Methods

We derived qualitative data primarily from population-level verbal autopsy narratives, programmatic reports and SMGL-related publications, and partner-specific evaluations that included focus group discussions and in-depth interviews.

#### Verbal Autopsy Narratives

Following the population-based household surveys, retrospective verbal autopsies were conducted and used to measure the medical causes and delay-associated factors of maternal deaths. An open-ended narrative was captured, detailing the experiences of women prior to maternal death and offering context to health facility results.

#### Programmatic Reports and Publications

We reviewed implementation partner reports and SMGL-related publications and evaluations primarily to describe the intervention activities that occurred under each strategy.^25^^,^^26^ When necessary, we contacted implementing partners for clarification to resolve discrepancies and to provide more in-depth descriptions of program activities. Data were used to outline SMGL activities and contextualize findings related to the second delay.

#### Partner-Specific Systematic Evaluations

Partners provided data from focus group discussions with community groups and in-depth interviews with health systems staff at the district and health facility levels to understand the perceived impact of interventions. We analyzed focus group discussions and in-depth interviews using content analysis.

From these qualitative data sources, we gathered information on the (1) description of strategies, (2) methods of implementation, (3) outputs (i.e., direct results of activities), and (4) outcomes (i.e., changes in knowledge or behaviors of the pregnant women/target population). Data were triangulated with the quantitative data to assess the implementation of strategies related to the second delay in the context of the SMGL initiative.

### Ethics

The study protocol was reviewed and approved by the ministries of health in Uganda and Zambia and deemed non-research by the Human Research Protection Office of the Center for Global Health at the U.S. Centers for Disease Control and Prevention. Written informed consent was obtained for respondents in all households and among women for the census, Reproductive Age Mortality Study interviews, focus group discussions, and in-depth interviews.

## RESULTS

This evaluation includes program data from multiple implementation partners that addressed the second delay to care within the context of the SMGL initiative. In this section, we first present the overall statistics for the change in rate of facility delivery within the SMGL districts by country. We then provide a brief description of the interventions under each SMGL strategy (Table 1) and their results.

The facility delivery rate at all facilities providing delivery services increased by 47% in Uganda and 44% in Zambia during the evaluation period (2011–2016). The increase in the facility delivery rate in Uganda was due to increased use of both facilities that met the EmONC requirements (45% increase) and of non-EmONC delivery facilities (49% increase) (Table 2). By contrast, the change in Zambia was primarily driven by increased use of non-EmONC delivery facilities (67% increase). The proportion of births in EmONC facilities increased to a lesser degree (12% increase) (Table 3). Facilities that did not meet BEmONC requirements may have had some, but not all, of the 7 interventions defining BEmONC.^2^

**TABLE 2.** Changes in Outputs and Outcomes Related to Activities Conducted Under SMGL Strategies Addressing the Second Delay in SMGL-Supported Districts, Uganda

	Baseline June 2012 (105 facilities)	Endline Dec 2016 (105 facilities)	% Relative change^a^	Significance level^b^
**Service delivery outcomes^c^**				
Deliveries in all facilities	45.5%	66.8%	+47%	*P*<.01
Deliveries in EmONC facilities	28.2%	41.0%	+45%	*P*<.01
Deliveries in non-EmONC facilities	17.3%	25.8%	+49%	*P*<.01
**Strategy 1: Decrease distance to skilled birth attendance by increasing the number of EmONC facilities^d^**				
Facilities offering services 24 hours a day, 7 days a week	80.0%	87.6%	+10%	NS
Facilities with electricity	57.1%	96.2%	+69%	*P*<.01
Facilities with running water	76.2%	100.0%	+31%	*P*<.01
Number of BEmONC facilities	3	9	+200%	NA
Number of CEmONC facilities	7	17	+143%	NA
Number of pregnant women who received antiretroviral therapy for the prevention of mother-to-child-transmission of HIV/AIDS	1,262	6,837	+442%	NA
Number of HIV-exposed infants receiving HIV prophylaxis	1,117	3,245	+191%	NA
Health facilities reporting that at least 1 doctor, nurse, or midwife is on staff	100.0%	100.0%	0%	NS
**Strategy 2: Improve the accessibility of EmONC facilities^d^**				
Institutional deliveries supported by Baylor transportation vouchers^e^	0.9%	23.8%	+258%	*P*<.01
Health facilities that reported having available transportation (motor vehicle or motorcycle)	61.0%	59.0%	−3%	NS
Health facilities that reported having communication equipment (including 2-way radio, landline, or cell phone with service)	93.3%	99.0%	+6%	*P*<.05

Abbreviations: BEmONC, basic emergency obstetric and newborn care; CEmONC, comprehensive emergency obstetric and newborn care; EmONC, emergency obstetric and newborn care; NA, not applicable; NS, not significant; SMGL, Saving Mothers, Giving Life.

a Percentage of change calculations are based on unrounded numbers.

b To test for significance, *z* scores based on the normal approximation to the binomial distribution were used to calculate *P* values.

c The number of facility deliveries was collected through the Pregnancy Outcome Monitoring data collection. The number of live births was estimated by applying crude birth rates (derived from the age-specific fertility rates among women of reproductive age enumerated in 2013 in the SMGL Uganda districts) to the baseline and endline district populations.

d The number of health facilities performing deliveries varied over the 5-year initiative. Health facility assessments results for Uganda were compiled from only the 105 facilities that maintained delivery capacity from baseline to endline.

e Transportation vouchers were introduced in April 2012 in the 3 Baylor districts; the system was rapidly scaled up with SMGL support.

**TABLE 3.** Changes in Outputs and Outcomes Related to Activities Conducted Under SMGL Strategies Addressing the Second Delay in SMGL-Supported Districts, Zambia

	Baseline June 2012 (110 facilities)	Endline Dec 2016 (110 facilities)	% Relative change^a^	Significance level^b^
**Service delivery outcomes^c^**				
Deliveries in all facilities	62.6%	90.2%	+44%	*P*<.01
Deliveries in EmONC facilities	26.0%	29.1%	+12%	*P*<.01
Deliveries in non-EmONC facilities	36.7%	61.1%	+67%	*P*<.01
**Strategy 1: Decrease distance to skilled birth attendance by increasing the number of EmONC facilities^d^**				
Facilities offering services 24 hours a day, 7 days a week	68.2%	96.4%	+41%	*P*<.01
Facilities with electricity	55.5%	92.7%	+67%	*P*<.01
Facilities with running water	90.0%	97.3%	+8%	*P*<.05
Number of BEmONC facilities	3	8	+167%	NA
Number of CEmONC facilities	4	5	+25%	NA
Number of pregnant women who received antiretroviral therapy for the prevention of mother-to-child transmission of HIV/AIDS	930	1,036	+11%	NA
Number of HIV-exposed infant receiving HIV prophylaxis	523	1,030	+97%	NA
Number of health providers hired	—	89	—	NA
Health facilities reporting that at least 1 doctor, nurse, or midwife is on staff	90.0%	98.8%	+10%	*P*<.05
**Strategy 2: Improve the accessibility of EmONC facilities^d^**				
Health facilities that reported having available transportation (motor vehicle or motorcycle)	55.5%	72.7%	+31%	*P*<.01
Health facilities that reported having communications equipment (including 2-way radio, landline, or cell phone with service)	44.6%	100.0%	+124%	NA
Health facilities that reported having an associated maternity waiting home	28.8%	48.8%	+69%	*P*<.01
Health facilities that reported having an associated Safe Motherhood Action Group	63.8%	96.3%	+51%	*P*<.01

Abbreviations: BEmONC, basic emergency obstetric and newborn care; CEmONC, comprehensive emergency obstetric and newborn care; EmONC, emergency obstetric and newborn care; NA, not applicable; NS, not significant; SMGL, Saving Mothers, Giving Life.

a Percentage of change calculations are based on unrounded numbers.

b To test for significance, *z* scores based on the normal approximation to the binomial distribution were used to calculate *P* values.

c The number of facility deliveries was collected through the Pregnancy Outcome Monitoring data collection. The number of live births was estimated by applying crude birth rates (derived from 2010 national census in Zambia) to the baseline and endline district populations.

d The number of health facilities performing deliveries varied over the 5-year initiative. Health facility assessments results for Zambia were compiled from only the 110 facilities that maintained delivery capacity from baseline to endline.

The facility delivery rate at all facilities providing delivery services increased by 47% in Uganda and 44% in Zambia during the evaluation period.

### Strategy 1: Decrease Distance to Skilled Birth Attendance by Increasing the Number of EmONC Facilities

Decreasing the distance to skilled birth attendance was addressed by upgrading a sufficient number of existing health facilities to meet BEmONC criteria in appropriate geographic positions (Table 1). One BEmONC criterion is to remain open 24 hours a day, 7 days a week with skilled staff present; this was a key element of the SMGL initiative to encourage and enable women to deliver in health facilities.^27^ Also critical for an EmONC facility is ensuring the availability of sufficient numbers of skilled birth attendants capable of managing complications (Table 1). During both Phase 1 and 2 of SMGL implementation, partners worked with ministries of health in both Uganda and Zambia to ensure facilities had staff capable of providing EmONC services.

#### Uganda

To address a shortage of health centers adequately equipped to handle deliveries, an implementing partner worked with districts to identify facilities that would benefit from additional support to enable them to provide BEmONC services in geographic areas lacking these services. Facilities previously offering only outpatient services, but which had adequate space, were supported with the necessary equipment and supplies to conduct delivery services, and skilled midwives were redeployed to work there. To ensure the availability of a sufficient number of skilled staff, medical officers, anesthetists, midwives, and nurses were hired and retrained in EmONC.

During the period of implementation, 12 health center II facilities (which are generally outpatient facilities) in hard-to-reach areas were strengthened to provide delivery services capable of managing basic complications. The number of EmONC facilities increased between baseline and endline by more than double—from 10 (3 BEmONC and 7 CEmONC) to 26 (9 BEmONC and 17 CEmONC), with a 200% increase in the number of health facilities capable of providing BEmONC services (Table 2). Though not statistically significant, Uganda saw a 10% relative increase in the proportion of health facilities offering services 24 hours a day, 7 days a week. At baseline, all SMGL facilities had at least 1 doctor, nurse, or midwife on duty and this remained the same at endline (Table 2).

An analysis of the changes in estimated travel time to reach EmONC facilities across SMGL time points in Uganda found that geographic access to BEmONC and CEmONC increased significantly (*P*<.01) within the 4 SMGL study districts between 2012 and 2016.^28^

#### Zambia

In Zambia, partners implemented a range of interventions including purchasing essential equipment, supplies, and medications necessary for EmONC, both basic and comprehensive; hiring additional midwives to fill existing vacancies; training doctors, midwives, and anesthetists in EmONC; and renovating health facility infrastructure, including making improvements to water source and provision of solar power when electricity was not available. Recently retired midwives were recruited to return to active service. In addition, implementing partners and district staff conducted monthly on-site mentorship of health facility staff using protocols, forms, and drills.

The number of EmONC facilities increased from 7 (3 BEmONC and 4 CEmONC) to 13 (8 BEmONC and 5 CEmONC). Zambia saw a 41% increase in the number of facilities offering services 24 hours a day, 7 days a week, and the proportion of health facilities reporting at least 1 doctor, nurse, or midwife on staff at the end of the project in 2016 improved significantly (*P*<.05) (Table 3). Specifics about health facility staff hires and trainings have been provided elsewhere.^27^

Zambia saw a 41% increase in the number of facilities offering services 24 hours a day, 7 days a week.

### Strategy 2: Improve the Accessibility of EmONC Facilities

As illustrated by an excerpt from a verbal autopsy (Box 1), the challenges of distance and transportation are substantial.

BOX 1 **Vignette Illustrating Challenges Related to Delay in Reaching Care, From a Verbal Autopsy**

Sylvia was a 23-year-old Ugandan woman who died giving birth to her third child, having had 2 previous births by cesarean delivery. Sylvia's father was interviewed during a verbal autopsy. The interview was transcribed and is summarized below. Details have been added in brackets to clarify meaning; names of people, places, and dates have been changed to protect confidentiality.

*At 5:00 a.m., Sylvia's father was called and told that his daughter needed help; she was in labor, which had started some hours earlier. He found her in serious pain and went to look for a motorcycle [to take her to a health facility]. By the time he got a motorcycle, Sylvia could not manage to sit on it. It had already started raining heavily. Sylvia's father contacted somebody who had a vehicle, but the driver told him he couldn't manage the trip because the road was impassible. The father contacted a second person with a vehicle and was again told the trip was not possible because of the poor condition of the road. Their village was about 10.5 km from the main road. The rain continued and at 11:00 a.m. Sylvia's brother came with a vehicle. By that time, the drug shop seller had put Sylvia on a drip [intravenous infusion] to stimulate contractions. While they were on the way to the health center number IV, or mini-hospital, the baby started bringing the head [crowning]. After they had been traveling for approximately 1 hour, Sylvia died before reaching the health center. They contacted the doctor to remove the fetus, but it had already died. The doctor told them that the uterus ruptured, which had caused Sylvia's death*.

In both Uganda and Zambia, a number of approaches were taken to improve access to EmONC services by establishing strong referral systems inclusive of communication and transportation. These approaches, detailed in the sections below, included strengthening maternity waiting homes, reinforcing communication and transportation systems, establishing community linkages to the health system, and facilitating better savings in preparation for delivery (Table 1).

#### Renovation and Construction of Maternity Waiting Homes

To increase access to EmONC services for women in need, partners renovated and constructed maternity waiting homes—residential lodging near facilities where women can stay while awaiting delivery—in both Uganda and Zambia. The temporary lodging spaces provided by maternity waiting homes enable health facilities with EmONC services to better accommodate mothers from hard-to-reach or distant communities who may otherwise experience transportation challenges at the time of delivery.

#### Uganda

In one district, a partner renovated maternity waiting homes at CEmONC hospitals, creating a waiting space for women at sites that were capable of providing comprehensive care services without needing to be referred elsewhere. During SMGL Phase 1, 4 maternity waiting homes were refurbished at 1 district hospital and 3 at EmONC-capable health center IVs (health centers that function as mini-hospitals). Newly renovated maternity waiting homes in Uganda accommodated approximately 10% of all mothers who delivered at the associated health facilities during Phase 1 and Phase 2 of the initiative.

Newly renovated maternity waiting homes in Uganda accommodated approximately 10% of all mothers who delivered at the associated health facilities in Phase 1 and Phase 2 of the initiative.

#### Zambia

In Zambia, some partners either constructed or renovated existing maternity waiting homes. As part of the Maternity Homes Alliance, other partners conducted formative research to design a community-informed maternity waiting home model during the end of SMGL Phase 1 and beginning of SMGL Phase 2 (2013–2014).^9^^,^^29^^–^^31^ During SMGL Phase 2 (July 2015), partners then refined the model with the government and constructed 24 maternity waiting homes in 7 SMGL districts across 3 provinces (Eastern, Luapula, and Southern) at sites where distance, physical geography, and terrain played a major role in determining access to EmONC services. Partners worked with health system staff, Safe Motherhood Action Group members, and traditional leaders to generate demand for maternity waiting homes. Beginning in SMGL Phase 2, partners began evaluating the impact of maternity waiting homes^32^ and assessing them for acceptability and sustainability.

During SMGL Phases 1 and 2, 211 maternity waiting home were either renovated (n=171) or newly constructed (n=40). Utilization data for all homes are not available, but in the 24 maternity waiting homes newly constructed by the Maternity Homes Alliance operating for the last 6 months of SMGL Phase 2, 1,123 women had used them before December 2016, approximately 49% of those delivering at the affiliated health facilities. Preliminary qualitative results from Zambia indicate that maternity waiting homes are acceptable to community members and that health facility staff perceive an increase in facility attendance for delivery and postnatal services (Box 2).

BOX 2 **Stakeholder Perceptions of Maternity Waiting Homes in Zambia**

*“It's always good to go and wait in the [maternity waiting home]. The doctors are always available and in case you have a complication, they always know fast. So that's why it's good to go and wait in the [maternity waiting home]*.”

—Focus group discussion with recently delivered or pregnant women

*“We are very happy because it used to be a problem for our children when they become pregnant; we would be very worried on where to take our children in case of delivery. But now that they have built a [maternity waiting home] which is very good and clean, we will be very free and happy to come and live here with our children*.”

—Focus group discussion with community elders

*“I think the appearance of the [maternity waiting home] is very good. The way I saw it … it really helps our women because everything is there. For a woman who is very pregnant, it's a very good thing*.”

—Focus group discussion with men

*“The success is that we no longer have mothers delivering from outside the facility, giving reasons that they were unable to come because they are coming from very far. Most of the mothers coming from distant places usually are admitted in our [maternity waiting home]. We have reduced on people having the excuse of delivery at home because of distance*.”

—In-depth interview with health facility staff

*“From the time the [maternity waiting home] was opened, we have seen that the number of women who are coming for deliveries has risen and the standard of the [maternity waiting home], which has been built now, is of high quality than the one we used to have, which was just a simple house and some women would not even want to stay in it*.”

—Focus group discussion with Safe Motherhood Action Group members

#### Communication and Transportation Services

A key element of the SMGL initiative was the creation of an integrated communication and transportation system that functions 24 hours a day, 7 days a week, to encourage and enable pregnant women to access delivery care facilities. Both Uganda and Zambia led several efforts to facilitate transportation to and between facilities.

#### Uganda

In Uganda, partners collaborated with the Ministry of Health to establish guidelines and referral procedures, which did not exist before Phase 1. The referral system consisted of 5 critical components (Box 3). A transportation committee was established in each SMGL-supported district that comprised the district health officer, assistant district health officer for maternal and child health, hospital superintendents, health center IV in-charges, ambulance drivers, and a project mentor midwife. These committees met monthly to review referrals and quarterly to review maternal and child health outcomes. The ambulance and referral systems were jointly coordinated by SMGL project staff and the district health office. To facilitate coordination, fixed phones were procured, enabling facilities to better communicate referrals with the district health office. The district staff communicated with the ambulance driver closest to the health facility, with clear instructions of the name of the facility needing the service and the name of the facility where the client was being taken. By phone, the district health office staff also provided mentorship on how to handle the patient as they waited on the ambulance to reach them.

BOX 3 **Details of the Ambulance Coordination Efforts in Uganda**

**Ambulance Coordination and Communication**

- Positioned tricycle and vehicle ambulances at strategic facilities for prompt referral
- Trained drivers in first aid and emergency care and provided first aid kits containing gloves and plastic sheets, surgical blades, cotton, and ligatures
- Availed contact lists for ambulance drivers at each health facility; these were networked with health facility and village health teams for toll-free calls (closed user group) to facilitate timely referral
- Referral calls received by a district health officer or senior midwife, including from private hospitals
- Monthly and quarterly committee meetings to review the number of referrals and outcomes, respectively, for quality improvement:
  - A total of 3,180 women in Phase 1 and 14,871 women in Phase 2 were transported by the ambulances for referral between facilities
  - When needed, senior midwives met with private and nonprofit hospitals to coordinate ambulances

**Ambulance Maintenance**

- Senior driver regularly checked fuel, tires, brakes, oxygen, and emergency supplies

**Human and Financial Resources**

- Around-the-clock (24 hours a day, 7 days a week) duty schedule and on-call sleep room for drivers at district hospitals
- Ambulance team included nurse-midwives, doctors, and emergency responders; picked up by drivers at night for emergency referrals
- Drivers hired by the Saving Mothers, Giving Life initiative who performed well were transitioned to government positions, as available

**Guidelines for Transport and Infection Control**

- Washing and disinfection of vehicles

**Referral Guidelines**

- Referral log book in triplicate: copy at referring site, copy at receiving site signed by attending midwife, and third copy in ambulance book
- Key vital signs recorded in log book
- Outcomes discussed in quarterly meetings

Secondly, partners in Uganda procured and distributed at least 1 ambulance to each SMGL district to supplement existing ambulances or fill a gap in districts with none. Large 4x4 vehicles were procured for areas with the most difficult terrain to navigate, smaller vehicles were procured for distant but easily navigable destinations, and motorized tricycle ambulances for areas that were nearer and had good terrain. The motorized tricycle ambulances were placed at the health sub-district or sub-county levels and the vehicles at the district level. This allowed the closest ambulance to the emergency to be assigned for timely referral of mothers and newborns with complications. SMGL partners supported existing ambulances within the districts with vehicle maintenance and repairs and by hiring and paying ambulance drivers' salaries and allowances. Program-based data included individual-level data such as the status of the patient, diagnosis, time of arrival, and reason for referral. These data were collected through referral forms completed at the destination health facility.

To facilitate transportation for women from the community to health facilities, Uganda implemented a “boda-for-mother” voucher program in 3 districts. This was guided by results from a health systems needs assessment conducted in April 2012, which indicated that *boda-bodas* (local motorcycles) were acceptable for transportation and could improve access to skilled birth attendance. Boda-bodas were engaged to facilitate the transportation of pregnant women from their villages to the nearest health facility providing EmONC as part of the voucher program. Transportation vouchers were distributed within the communities by village health team members to ensure women's access to health facilities and to reach upper-level referral facilities in the event of a delivery-related emergency. Village health teams are community volunteers affiliated with health facilities and engage during health promotion activities at the community level.^33^ The transportation vouchers were expanded during Phase 2 to provide transportation not only for delivery but also for 4 antenatal care visits and 1 postnatal care visit. Thirty percent of transportation vouchers were redeemed, resulting in a 258% increase (P<.0001) in the proportion of deliveries supported by boda-for-mother transportation vouchers (Table 2).

Although the percentage of facilities reporting the availability of motor vehicle transportation was stable in Uganda (61% at baseline and 59% at endline), there was a 6% increase (*P*<.05) in the percentage of health facilities that reported having communications equipment (Table 2).

#### Zambia

Zambia had existing referral guidelines and procedures before Phase 1, consisting of triplicate Ministry of Health referral forms or books that logged the time the transportation was called, time of patient pickup, time of arrival to hospital, outcome of mother and baby, and feedback to the referring facility. SMGL partners strengthened the use of existing referral procedure guidelines and supported printing of the triplicate referral forms and log books. Over time, support for the printing of log books was withdrawn and districts took on the printing of referral logs for their facilities. Technical committees met monthly to review transportation coordination, patient referrals, partner coordination, and other maternal health issues. Program-based data included referral forms and logs in each facility and at the district level. A pilot program in Kalomo District conducted in 2012–2013 used a transportation checklist to help stabilize pregnant women before moving them to a higher-level facility for emergency procedures or surgery; this strategy was not scaled up beyond Kalomo and was not rigorously evaluated.

Similar to Uganda, ambulances were procured in Zambia to supplement existing ambulances in SMGL districts. The need for ambulances was identified through updates at provincial and district-level monthly meetings, and districts (through SMGL partner organizations) procured ambulances to fill the identified gaps. The strategic placement of ambulances within the districts was dependent on availability and the most efficient distribution. Ambulances were coordinated by district transportation committees. In Lundazi District, for example, where travel time from facilities to the district hospital is about 6 hours during the rainy season, the district positioned ambulances at strategic health facilities, so they would need to go only in one direction when referral to the hospital was needed. In Mansa District, on the other hand, the placement of ambulances was zonal. Mansa District is divided into 5 zones and each zone has a central “zonal” health facility (with higher-level services) that serves all health centers within that zone. The 3 ambulances procured under the SMGL initiative were placed in 3 of the 5 zones that did not already have an ambulance. The district transportation committees (a subset of the district technical committee) were responsible for the coordination of ambulance services. To request an ambulance, health facilities communicated with committee members by phone or radio messaging.

Bicycle ambulances (*Zambulances*) and motorcycle ambulances were procured in Zambia to provide transportation for pregnant women from the community to the health facilities. In some instances, the motorcycle ambulances were used for transportation of referral cases from health facilities to higher-level facilities or hospitals, filling the gap of unavailable motor vehicle ambulances. Safe Motherhood Action Group members were trained as motorcycle riders and worked as volunteers.

Lastly, to facilitate transportation between facilities, radios were repaired, and, where needed, cell phones or talk time were provided to enable communication between facilities and districts to improve coordination of ambulance services.

The availability of motor vehicle transportation improved significantly (*P*<.01) in Zambia, and there was a 124% increase in facilities that reported having communication equipment (Table 3). SMGL partners procured and distributed 1,500 bicycle ambulances; however, partner reports indicate this intervention was not successful because the bicycle-drawn carriage was an uncomfortable mode of transportation for pregnant women.

The availability of motor vehicle transportation improved significantly in Zambia, and there was a 124% increase in facilities that reported having communication equipment.

#### Community-Based Linkages to the Health Facility

In addition to the transportation schemes, some programs facilitated community health facility linkages in both Uganda and Zambia.

#### Uganda

In Uganda, using the Ministry of Health CHW training manual, village health team members were trained on maternal and newborn health issues. Within the communities, the village health teams distributed transportation vouchers and facilitated communication with the ambulance coordination team to transport women who had complicated pregnancies to health facilities. In addition, during Phase 2 only, in response to requests from the community, portable stretchers were procured and distributed to communities with terrain inaccessible by both vehicles and motorcycles. These were used to transport pregnant women or sick people to pickup points (by either the boda or ambulance vehicles) or health facilities.

#### Zambia

In Zambia, a cadre of non-clinical, community-based Safe Motherhood Action Group volunteers was expanded and trained extensively in safe motherhood strategies. This group had been supported initially on a pilot basis by a few nongovernmental organizations to help facilitate access to skilled deliveries. Safe Motherhood Action Group members were trained to educate women and their families about the risks associated with giving birth at home and with labor complications, and encourage them to develop birth plans, attend antenatal care, and give birth in a facility. In addition to the role they served addressing the first delay, the members also escorted women to the facility for delivery and in some instances called facilities to facilitate transportation of women from the community to the facility.^34^ Working in the community with direct links to the health facility, Safe Motherhood Action Group members were provided with mobile phone minutes or “airtime” to call the facility or call for transportation in an emergency. The proportion of health facilities that reported having an associated Safe Motherhood Action Group increased by 51% (*P*<.01) in Zambia (Table 3).^33^

#### Savings for Delivery as Part of Birth Preparedness

#### Zambia

To address costs associated with access to delivery service, even when the delivery service itself is free of charge, different models of saving for birth preparedness were implemented at the community level in Zambia during the latter part of SMGL Phase 2. One partner tested a variety of savings groups approaches across villages in Choma and Kalomo districts, to assess the most effective model of community savings using a training-of-trainers approach. Safe Motherhood Action Groups were trained on the savings models and they in turn worked with the community savings groups to guide selection of a savings model and provide oversight for the groups. Another partner working in Mansa, Chembe (Chembe District was part of Mansa District during Phase 1), and Lundazi districts worked with Savings and Internal Lending Community (SILC) groups, which were developed as a strategy to provide low-income people, especially women, access to resources for income-generation opportunities through loans from self-managed savings.^35^ With a membership of 15 to 20 people, each person saved an equivalent of US$5 in a general pool, from which members borrowed loans at an interest rate of 10% to 25% per loan or per month, depending on what was set out in the group constitution. Members also contributed to a social fund from which women drew money for costs associated with access to delivery services such as transportation to the health facility, baby clothes, and supplies needed for delivery. Women of reproductive age were mobilized into the SILC groups as a mode of saving for delivery.

Preliminary results show that through the training-of-trainers model, savings have been integrated into home-based counseling for birth preparedness, and village savings groups have incorporated new mechanisms into their savings group constitution to enable women to save for the costs associated with delivery, such as transportation to a health facility, delivery supplies, and baby clothes.^9^ Nearly all (96%) of the savings groups are offering loans to pregnant women at reduced interest rates (median 5% for pregnant women and 20% for other group members), 10% are offering 0% interest loans for pregnant women, 87% have a provision to offer a bonus (median US$2) to pregnant women who demonstrate preparedness for delivery, 50% have a maternity fund focusing specifically on maternal services, and 100% have a provision for pregnant women to store their money in the group's lockbox.

Saving for delivery has been integrated into home-based counseling for birth preparedness in Zambia, and village savings groups have helped enable women to save for costs associated with delivery.

The 319 SILC groups supported in Mansa, Chembe, and Lundazi had a total of 6,862 members. Of group members, 74% were women of reproductive age. Members of the SILC groups feel more prepared for delivery, as explained by a member of the group:

Through SILC I managed to buy all necessities for my baby and myself. I went and delivered a bouncing baby girl at the health center. Through SILC, I was able to prepare for transport to take me to the health facility on time.

—SILC member, recently delivered woman

## DISCUSSION

In Uganda and Zambia, SMGL employed 2 key strategies to improve a woman's ability to reach EmONC services and ultimately improve maternal and newborn outcomes. These strategies addressed the known causes of delays in reaching care including distance, geography, accessibility, lack of transportation and communication, and costs associated with delivery.^5^^,^^6^^,^^36^ Under each strategy, a set of interventions was implemented to address the second delay. Strategy 1—interventions to increase the number of EmONC facilities—primarily included upgrading strategically positioned health facilities to be capable of providing EmONC services and providing EmONC trainings and in-service mentorship for health staff. Strategy 2—interventions to improve the accessibility of EmONC facilities—included renovations and construction of maternity waiting homes, creation of integrated communication and transportation systems, establishment of community-based linkages to the health facility, and programs to encourage savings for delivery as part of birth preparedness. Though it is difficult to disentangle the effects of each intervention within the context of complex, multilevel programs, it is reasonable to conclude that collectively, these interventions addressed challenges associated with the delay in reaching care in both Uganda and Zambia.

### SMGL's Successes

SMGL's comprehensive approach of targeting all 3 delays is likely more programmatically meaningful than tackling interventions focusing solely on a single delay. In both countries, the proportion of facility deliveries between 2012 and 2016 in SMGL-supported districts increased significantly. An analysis of delivery location among women living in remote Zambia found that those living in districts unexposed to the SMGL initiative were 3 times more likely to deliver at home compared with those living in SMGL districts—offering additional evidence to support the benefits of this initiative.^12^ In a separate analysis of household-level data on women's reported place of birth in 1 district in Zambia between 2011 and 2013, women in SMGL districts had a 45% increase in the odds of facility delivery after the program was implemented relative to a comparison group within the same province with no SMGL exposure,^37^ suggesting that the rapid increase was not attributable solely to other contextual factors.

SMGL second-delay interventions in both countries related to communication and transportation focused on key elements of referral systems, including decreasing distance to skilled birth attendance, improving transportation, strengthening facility capacity to manage complications (e.g., EmONC), and establishing community linkages. Referral and transportation strategies alone have been estimated to account for an 80% reduction in maternal mortality.^38^ A systematic review of the referral-based interventions that were addressed in the second delay generally found that most interventions improved utilization of maternity care.^39^ Another review of referral systems in maternity care cited the need for appropriate communication and transportation, both of which were addressed by the SMGL initiative's second-delay strategies, as well as for appropriate protocols and monitoring of staff performance.^40^ Recent studies have also demonstrated the effectiveness of having community health workers use mobile phones to reduce delays in seeking and accessing care, improving health education and promotion, and facilitating timely referrals,^41^ a strategy similar to that employed by SMGL's community-based linkages interventions.

The provision of transportation vouchers for maternity care has also gained traction recently; this approach was a pillar of SMGL work in Uganda and is being replicated as a national program. The use of transportation vouchers in Uganda as a mechanism for improving transportation of mothers from the community to the health facility was key to increasing facility deliveries.^42^ In general, voucher programs have been shown to be effective at improving utilization of health care services, though there remains little to no evidence of improvement of quality of care or health outcomes.^18^ Previous research from SMGL Phase 0 indicated that engaging private-sector transportation providers was an important feature and that the availability of transportation made a large difference in increasing access for maternity care.^43^ SMGL interventions aimed to tackle equity through vouchers in Uganda and availability of transportation in both Uganda and Zambia.

As part of its strategy to improve the accessibility of EmONC facilities, SMGL implementing partners and collaborators refurbished or constructed maternity waiting homes in both countries. Though there is limited rigorous data, evidence suggests that higher-quality maternity waiting homes are associated with higher rates of facility deliveries.^44^ Additionally, a qualitative analysis across 17 countries found that barriers to utilization of maternity waiting homes included a lack of knowledge, poor structures, and too little space; the SMGL collaborators implemented maternity waiting homes that were designed with community input as part of a health system intervention.^45^ Maternity waiting homes represent a promising strategy to address the second-delay barriers and are being more comprehensively evaluated in this context.

SMGL interventions included strengthening a cadre of community-based health workers in each country—village health teams in Uganda and Safe Motherhood Action Groups in Zambia. In addition to helping address the first-delay challenges,^33^ these cadres played critical roles by supporting the referral system, distributing vouchers, and escorting women to facilities. A Cochrane review evaluating the effectiveness of community-based programs on maternal and newborn health found that community health workers can have positive impacts on increased facility-based delivery.^46^ Additionally, a qualitative study in Zambia found that Safe Motherhood Action Group members are perceived to have a positive impact on facility delivery and utilization of maternity waiting homes.^34^

Lastly, the savings for delivery strategies, implemented primarily in Zambia, may be effective interventions for reducing delays in accessing care. Better planning for delivery has been shown to increase uptake of antenatal care services and facility delivery in Tanzania.^47^ Additionally, as part of birth preparedness, savings for delivery may empower women to overcome distance, transportation, and supply challenges.^48^

Though it is challenging to isolate the specific contribution of individual programmatic elements, taken together, the intensive, multifaceted strategies of the SMGL initiative tackled many of the factors fueling the second delay and thereby contributed to an increase in facility delivery rates in both Uganda and Zambia. These efforts alone may not necessarily have improved health outcomes; however, combined with the SMGL efforts targeting the first and third delays, SMGL efforts can be deduced to have contributed to both increasing maternity care utilization and improving key health outcomes. ^23^^,^^24^

### Limitations

There were several limitations to understanding the effects of the SMGL initiative in both Uganda and Zambia. First, there was no comparison with non-implementing districts, thus making it difficult to assess the overall impact of the initiative. Second, within the SMGL districts during Phase 1, routine data collection systems were not harmonized across partner organizations, making it difficult to aggregate indicators to allow assessment of the impact of interventions. A more coordinated and systematic program evaluation effort integrated from program inception would have allowed for a better assessment of program effects.

In addition to the challenges around evaluation, 2 notable implementation limitations may impact program sustainability. First, increasing the availability of ambulances to facilitate referrals was undoubtedly key for improving access to health facilities. However, high costs including providing 24-hour driver coverage and fuel may limit the availability of ambulance services after SMGL. In some cases these high costs led to the transfer of fuel costs to women and their families who were often not able to cover the cost. Second, it was not always possible for a health facility staff member to accompany and monitor women during the referral journey. Finally, in Uganda, a high demand for transportation vouchers meant the vouchers were not always available to women in need. Village health teams who sold the vouchers did not always adhere to eligibility criteria, and some boda-boda drivers procured vouchers for resale at higher prices, often resulting in inequitable distribution of vouchers.

## CONCLUSION

Approaches outlined in this article to address the second delay (ability to reach care) can be adapted in low-resources settings to improve access to maternity care services and aim to reduce maternal and perinatal death. Through the SMGL initiative, multiple strategies were implemented to address the second delay, including increasing the number of BEmONC-equipped facilities and improving access to EmONC through improved systems of transportation and communication, temporary lodging in maternity waiting homes, and community-based savings groups. Collectively, these strategies resulted in increased access to skilled delivery services. There is a need to sustain and improve on these efforts to maintain and further address factors that influence a woman's ability to reach care in Uganda and Zambia.

## Notes

**Peer Reviewed**

**Funding:** Saving Mothers, Giving Life implementation was primarily funded by the Office of the Global AIDS Coordinator, the U.S. Agency for International Development, Washington, D.C., the U.S. Centers for Disease Control and Prevention, Atlanta, Georgia (Cooperative agreements GPS003057 and GPS002918), Merck for Mothers, and Every Mother Counts. The funding agencies had no influence or control over the content of this article.
